# Robotic cytoreductive surgery and hyperthermic intraperitoneal chemotherapy: is there a benefit?

**DOI:** 10.1007/s00464-024-11199-7

**Published:** 2024-10-16

**Authors:** Brian K. Sparkman, Devon C. Freudenberger, Vignesh Vudatha, Jose G. Trevino, Adam Khader, Leopoldo J. Fernandez

**Affiliations:** 1 Division of Surgical Oncology, Department of Surgery, Virginia Commonwealth University School of Medicine, 1200 E Broad St, PO Box 980011, Richmond, VA 23219 USA; 2 Department of Surgery, Richmond Veteran Affairs Medical Center, 1201 Broad Rock Blvd, Richmond, VA USA

**Keywords:** Robotic surgery, Minimally invasive surgery, Cytoreductive surgery, Peritoneal metastasis, Carcinomatosis, HIPEC

## Abstract

**Background:**

Open cytoreductive surgery (CRS) with hyperthermic intraperitoneal chemotherapy (HIPEC) is a therapeutic option for the management of malignancies with peritoneal carcinomatosis and of peritoneal origin. Robotic surgery shows promise as a minimally invasive approach for select patients. We aimed to evaluate the differences in outcomes between robotic versus open CRS/HIPEC and hypothesized less morbidity and faster recovery in the robotic approach group.

**Methods:**

We conducted a retrospective cohort study from our HIPEC database including all tumor origins. We included patients aged 18–89 years who underwent CRS/HIPEC for curative intent at a single institution between January 1, 2017, and December 31, 2023. Patients were stratified by open versus robotic-assisted surgery. Mann–Whitney U and Fisher Exact tests were used to compare differences in patient characteristics and outcomes.

**Results:**

A total of 111 patients underwent CRS/HIPEC for curative intent, with 95 (85.6%) cases performed open and 16 (14.4%) robotically. The groups were demographically similar, except patients undergoing robotic CRS/HIPEC had a significantly higher median income ($83,845 vs. $70,519, *p* < 0.001). Rate of comorbidities and cancer type, including appendiceal, colorectal, and ovarian, were the same. The peritoneal carcinomatosis index and completion of cytoreduction score were similar between groups. Robotic approach was associated with statistically significant lower estimated blood loss (113 vs. 400 mL, *p* < 0.001) and postoperative transfusions (6.3% vs. 23.2%, *p* = 0.036). Total complications, readmission rates, and 30-day mortality were similar among groups, but the robotic group had a significantly shorter length of stay (5.5 vs. 9 d., *p* < 0.001).

**Conclusion:**

Robotic CRS/HIPEC holds promise to decrease intraoperative blood loss, blood transfusions, and hospital stay while providing similar immediate postoperative outcomes in select patients. These results should be validated in the setting of a prospective trial and effects on long-term oncologic outcomes should be investigated.

Cytoreductive surgery (CRS) with hyperthermic intraperitoneal chemotherapy (HIPEC) is a major abdominal surgery for select patients with peritoneal carcinomatosis that carries significant morbidity and mortality and is associated with extended hospital stays of nearly two weeks [1, 2]. Open CRS/HIPEC is the most common technique. However, like other abdominal surgeries, minimally invasive techniques are being investigated [1]. Both laparoscopic and robotic-assisted minimally invasive approaches have progressed dramatically since their inception. As minimally invasive surgery is becoming more commonplace, more complex procedures are being attempted. Today, oncologic operations such as radical hysterectomies, colectomies, and cytoreductive radical prostatectomies are performed with increased safety and faster patient recovery while maintaining oncologic standards for select patients [3, 4].

CRS/HIPEC is regarded as the standard of care for pseudomyxoma peritonei [5]. In addition to pseudomyxoma peritonei, CRS/HIPEC is utilized for various peritoneal surface malignancies (PSM), such as metastatic gastrointestinal and gynecological cancers limited to the peritoneum [6–10]. Peritoneal metastasis can be quantified via the peritoneal cancer index (PCI). It is a scoring system that assesses the volume and distribution of peritoneal disease and provides a value to gauge disease burden with a range of 0–39 [11]. Depending on the origin of tumor, different PCIs may be appropriate for CRS/HIPEC. Achieving a complete cytoreduction, or removal of all gross disease, is important as it offers a survival advantage when combined with HIPEC over systemic chemotherapy [12, 13]. Open cytoreduction surgery is the gold-standard exposure for stripping the diseased peritoneum and achieving complete cytoreduction. However, open CRS/HIPEC has been associated with a 10–30% incidence of grade III/IV morbidities, similar to other complex oncologic abdominal surgeries such as hepatectomy or pancreaticoduodenectomy [14]. The median hospital stay is 10 to 14 days [1, 14]. Laparoscopic CRS/HIPEC has been performed for select patients providing favorable oncologic outcomes and it has shown decreased intraoperative blood loss, hospital stay, and time to adjuvant chemotherapy [15, 16]. Patient-related experience measures, such as changes with work or exercise, have shown minimal change after a laparoscopic CRS/HIPEC compared to pre-operative experiences [17].

Multiple studies have described laparoscopic approaches to CRS/HIPEC in patients with low burden disease defined by a PCI of less than 10. However, few have evaluated robotic-assisted approaches [18, 19]. As robotic-assisted surgery becomes more available and familiar to surgeons, it will likely become a more common approach for CRS/HIPEC. Due to its improved visualization, stability, and dexterity a robotic-assisted approach may increase the chance of complete cytoreduction via a minimally invasive approach, potentially expanding the pool of patients with higher disease burden. Herein we detail our approach for robotic-assisted CRS/HIPEC. Further, we compare our postoperative outcomes of patients with robotic-assisted to open CRS/HIPEC. We hypothesized that patients undergoing robotic-assisted CRS/HIPEC would have decreased morbidity, faster recovery, and decreased blood loss while still achieving completeness of cytoreduction compared to patients undergoing open CRS/HIPEC.

## Materials and methods

### Patient selection and clinical data

A retrospective review of a prospectively maintained database was performed at a single institution for patients who underwent CRS/HIPEC from January 1, 2017, to December 31, 2023. Patients were included if they were 18–89 years old and undergoing CRS/HIPEC for curative intent. Patients who underwent HIPEC without CRS, such as for palliation of ascites, were excluded. CRS was performed with curative intent to remove all peritoneal disease to achieve a complete cytoreduction. HIPEC was performed with chemotherapy choice and duration of therapy dictated by the individual patient’s cancer type. All data were collected from electronic medical records. This study was approved by the Virginia Commonwealth University Institutional Review Board (Protocol Number: HM20029639).

Clinical data included patient sociodemographic factors, comorbidities, oncologic history, intraoperative and postoperative outcomes. Sociodemographics included patient age, sex, self-reported race/ethnicity, insurance payor, distance traveled to the hospital (obtained by calculating the distance from the patient’s listed zip code city center to the treating medical center), and median household income (obtained using the patient’s listed zip code and US Census Data from the American Community Survey 5-year estimates from 2015 to 2019).

Specifically recorded patient comorbidities included presence of hypertension, diabetes, chronic obstructive pulmonary disease (COPD), coronary artery disease, chronic kidney disease, and current smoking status. Additionally, the patient’s preoperative American Association of Anesthesiologist Physical Status Classification System score (ASA score) was noted.

Intraoperative data points included the length of surgery (minutes), surgeon calculated peritoneal cancer index (PCI), completion of cytoreduction score (CC score; a standardized measure of the amount of disease remaining after cytoreduction), intraoperative blood transfusion, estimated blood loss (EBL), and creation of an ostomy. Postoperative variables included minor complications (Clavien-Dindo classification types I-II) and major complications (Clavien-Dindo classification types III-IV) within 30 days of surgery, length of hospital stay, readmission within 30 days of surgery, and 30-day mortality.

### Statistical analysis

Data were stratified by surgical approach: open versus robotic. Given the non-parametric distribution of data, differences between surgical approach for patient sociodemographics, comorbidities, intraoperative and postoperative outcomes were compared using Mann–Whitney U Test for continuous variables and Fisher’s Exact test for categorical variables. Tests were two-sided with an alpha value of 0.05 indicating significance. All data analyses were completed using IBM SPSS 28.0.0 (Armonk, NY).

### Description of surgical procedures

Before selecting between a robotic-assisted or open cytoreduction, patients are staged and evaluated to establish burden of disease based upon their underlying tumor type. This includes an upper and/or lower endoscopy, computed tomography of the chest, abdomen, and pelvis, and a diagnostic laparoscopy for direct visualization of the peritoneum and establishment of a PCI, and determination of the location of disease. We do not use a PCI cutoff to exclude patients from robotic-assisted cytoreduction, but rather patients are assessed for the feasibility of achieving a complete cytoreduction based on location and burden of disease and estimation of an operative reasonable time. After staging is complete, patients are stratified to an open or robotic approach.

All patients are positioned in low lithotomy with arms out. Central venous access is obtained and an arterial line established. Preoperative antibiotics are given and redosed throughout the case. A temperature-sensing Foley catheter is placed sterilely. A diagnostic laparoscopy is performed to confirm stability and resectability of disease prior to committing to CRS/HIPEC.

### Open CRS/HIPEC

Open CRS/HIPEC is performed via a midline laparotomy. The PCI is systematically calculated and resection planned. Peritonectomy of the affected serosal surfaces and visceral resections are performed. Once complete, HIPEC is delivered via closed technique. Intra-abdominal chemotherapy is administered according to established protocols. After completion of HIPEC, the chemoperfusate is drained and the abdomen is rinsed with saline. The cannulas are removed and, if necessary, alimentary anastomoses are performed. The abdomen is inspected for any injuries and repaired if identified. Drains are placed as indicated. A nasojejunal feeding tube is advanced into position, as is a nasogastric tube. The abdomen is closed in layers and dressings applied.

### Robotic CRS/HIPEC

**Fig. 1:**
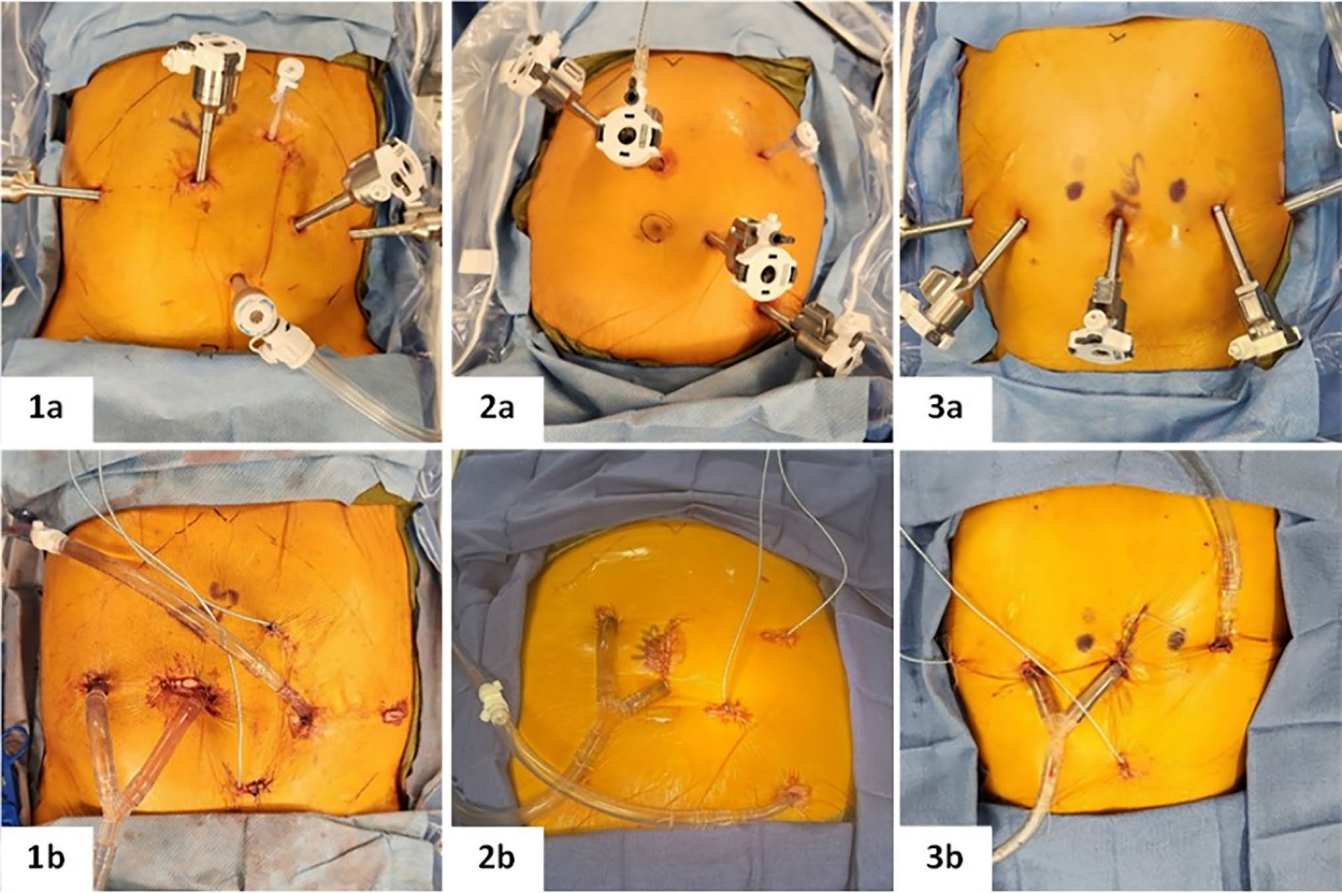
Port and chemoperfusion cannula placement for robotic-assisted CRS/HIPEC. Our standard robotic-assisted CRS/HIPEC setup (1a, 1b). Robotic trocars are placed flat across the abdomen eight centimeters apart. However, variations on the setup (2a/2b, 3a/3b) are utilized for optimal positioning

Insufflation is established with a Veress needle to 15 mmHg. A robotic trocar is placed via an optiview technique, and a diagnostic laparoscopy is performed. If deemed adequate for robotic cytoreduction, three additional robotic trocars are placed flat across the abdomen, spaced 8–10 cm apart, with two ports placed to the left of the umbilicus and one to the right. Assistant ports are placed in the cephalad and caudal portions of the abdomen to assist with insufflation, hemostasis with an energy device, suture insertion, stapling, specimen removal, suction, and retraction (Fig. 1). The patient is then positioned in Trendelenburg at approximately 15 degrees. Starting in the pelvis, we confirm our disease burden and perform cytoreduction. Once the pelvis and distal small bowel are completely cytoreduced we then undock the robotic arms. The patient is positioned in mild reverse Trendelenburg and the robot is redocked with the arms positioned for the upper abdomen. Cytoreduction of the upper abdomen, and proximal jejunum are completed. We then perform the omentectomy, with hook cautery and bedside assistance with an advanced energy device. The assistant port is also used for stapler insertion for any resections requiring stapling. The lesser omentum is opened allowing complete examination of the caudate lobe, ligamentum venosum, and left side of the inferior vena cava. The falciform and ligamentum teres are completely excised. Close attention is taken to examine all the areas of the abdomen and avoid missing any lesions. After cytoreduction, all specimens are placed within specimen bags and left in the abdomen until the robot is undocked. The abdomen is deflated and the specimens are retrieved after the enlargement of the supraumbilical incision to facilitate retrieval. Bowel anastomoses are performed at this time as indicated. Esophageal and rectal anastomosis, if indicated, are performed after chemoperfusion. The abdomen is irrigated with 3 L of normal saline to remove large particulate that may interfere with flow of the perfusion circuit. The Lasso outflow cannula is inserted at the umbilicus, brought out via a right sided trocar site and positioned above the liver laparoscopically. Trocar sites are used for the inflow cannula and positioned within the pelvis (Fig. 1). Two temperature probes are placed intraperitoneally. Insufflation is re-established and cannula placement is confirmed. Chemoperfusion is then performed. Afterward, the abdomen is drained and the cannulas are removed under laparoscopic visualization. The abdomen is inspected for any injuries that may have occurred and repaired. A drain is placed within the dependent portion of the abdomen. A nasogastric tube is positioned within the stomach. The fascia is closed for all port sites and incisions greater than 1 cm. All incisions are closed at the skin and tissue adhesive is applied.

## Results

Over six years, 111 patients underwent CRS/HIPEC for curative intent. Of these 95 (85.6%) cases were performed open and 16 (14.4%) were performed robotically (Table 1).

**Table 1:** Patient demographics for open versus robotic CRS/HIPEC. Categorical variables are presented as n (%) and continuous variables are presented as median [interquartile range]

Variable	Open (*n* = 95)	Robotic (*n* = 16)	*p*-value
Sex			0.585
Male	39 (41.1)	5 (31.3)	
Female	56 (58.9)	11 (68.8	
Age (y)	58 [49, 66]	54 [42, 70.5]	0.442
Race			0.301
Asian	3 (3.2)	0 (0.0)	
Black	28 (29.5)	2 (12.5)	
Other	6 (6.3)	0 (0.0)	
White	58 (61.1)	14 (87.5)	
Ethnicity			1.000
Hispanic	9 (9.5)	1 (6.3)	
Non-hispanic	86 (90.5)	15 (93.8)	
Distance traveled	18.9 [12.5, 53.8]	31.7 [18.4, 66.7]	0.072
Median income	70,519 [50,559, 79,692]	83,845 [72,503, 96,428]	< 0.001
Insurance			0.534
Medicaid	12 (12.6)	1 (6.3)	
Medicare	28 (295)	3 (18.8)	
Private	52 (54.7)	11 (68.8)	
Tricare	2 (2.1)	1 (6.3)	
Uninsured	1 (1.1)	0 (0.0)	
Insurance			0.500
Govt	42 (44.2)	5 (31.3)	
Private	52 (54.7)	11 (68.8)	
Uninsured	1 (1.1)	0 (0.0)	
Comorbidities			
CAD	7 (7.4)	2 (12.5)	0.615
HTN	50 (52.6)	7 (43.8)	0.594
COPD	2 (2.1)	0 (0.0)	1.000
DM	17 (17.9)	1 (6.3)	0.462
CKD	2 (2.1)	1 (6.3)	0.376
Smoker	6 (6.3)	1 (6.3)	1.000
ASA			0.514
2	11 (11.6)	3 (18.8)	
3	79 (83.2)	13 (81.3)	
4	5 (5.3)	0 (0.0)	
BMI	30.6 [26.6, 34.4]	27.9 [23.8, 31.6]	0.098
Primary cancer			0.270
Appendiceal	35 (36.8)	8 (50.0)	
Colorectal	30 (31.6)	3 (18.8)	
Other^a^	18 (18.9)	1 (6.3)	
Ovarian/fallopian	12 (12.6)	4 (25.0)	

^a^Other includes diagnoses of adrenocortical, esophageal, gallbladder, gastric, mesothelioma, sarcoma, small bowel, unknown, and urachal primary cancers

When comparing patient demographics there was no significant difference in patient age, sex, race or ethnicity, or insurance payor between the two groups. However, patients who underwent robotic approach had significantly higher median income ($83,845 vs. $70,519, *p* < 0.001) and trended toward traveling further to receive care (31.7 vs. 18.9 miles, *p* = 0.072) compared to those who underwent open surgery. Patients also had similar rates of comorbidities and ASA score, though patients in the robotic group trended toward having a lower BMI than those in the open group (27.9 vs. 30.6 kg/m^2^, *p* = 0.098). Additionally, there were similar rates of appendiceal, colorectal, gynecologic, and other cancer types treated in both groups.

As indicated by the surgeon scored PCI and CC-score, patients in the open and robotic groups had similar burdens of disease and in most cases achieved a CC-score of 0 (Table 2). Ascites was encountered exclusively in the open group (11.6 vs. 0%, *p* = 0.360) though this was not significant. Bowel anastomoses were more often performed in patients undergoing open surgery compared to robotic (66.3 vs. 43.8%, *p* = 0.099). Importantly, the robotic group had significantly less median estimated blood loss than the open group (113 vs. 400 mL, *p* < 0.001). Despite not reaching significance, intraoperative blood transfusions occurred nearly four times more often in patients undergoing CRS/HIPEC (23.2 vs. 6.3%, *p* = 0.185) than in patients in the robotic approach. The total operative time was similar between groups.

**Table 2:** Intraoperative outcomes for patients undergoing open versus robotic CRS/HIPEC. Categorical variables are presented as **n** (%) and continuous variables are presented as median [interquartile range]

Variable	Open (*n* = 95)	Robotic (*n* = 16)	*p*-value
PCI	12 [8, 20]	10 [7, 16]	0.264
CC score			1.000
0	83 (89.2)	15 (93.8)	
1	10 (10.8)	1 (6.3)	
Ascites	11 (11.6)	0 (0.0)	0.360
EBL (mL)	400 [300, 800]	113 [85, 188]	< 0.001
Intraoperative time (minutes)	565 [452, 721]	617 [510, 804]	0.187
Intraoperative blood transfusion	22 (23.2)	1 (6.3)	0.185
Ostomy creation	11 (11.6)	1 (6.3)	1.000
Bowel anastomosis performed	63 (66.3)	7 (43.8)	0.099

Postoperatively, 32.6% of patients in the open surgical approach required a blood transfusion, whereas only 6.3% of patients in the robotic group required a blood transfusion, which was statistically significant (*p* = 0.036) (Table 3). Though total complications were similar between the groups, patients in the robotic group had fewer minor complications than the open group (*p* = 0.030). Length of stay was nearly halved in the robotic group with a median of 5.5 days compared to 9 days in the open group (*p* < 0.001). Rates of readmission were less than 20% in both groups (*p* = 0.732). Of the 111 cases, there was only one 30-day mortality (0.9%).

**Table 3:** Postoperative outcomes for patient undergoing open versus robotic CRS/HIPEC. Categorical variables are presented as n (%) and continuous variables are presented as median [interquartile range]

Variable	Open (*n* = 95)	Robotic (*n* = 16)	*p*-value
Blood transfusion	31 (32.6)	1 (6.3)	0.036
Ileus	27 (28.4)	4 (25.0)	1.000
Surgical site infection	6 (6.3)	0 (0.0)	0.591
Minor complications	1 [0, 2]	0 [0, 1]	0.030
Major complications	0 [0, 0]	0 [0, 0]	0.634
Total complications	1 [0, 2]	0 [0, 1]	0.105
Length of stay (days)	9 [6, 12]	5.5 [4, 7]	< 0.001
Readmission within 30 days	18 (18.9)	2 (12.5)	0.732
30-day mortality	1 (1.1)	0 (0.0)	1.000

## Discussion

Prior studies have demonstrated that laparoscopic and robotic-assisted surgery maintains perioperative safety for operable tumors comparable to open resection [20]. Laparoscopic CRS/HIPEC has been shown to provide equivalent perioperative and oncologic outcomes as compared to open CRS/HIPEC for select patients [15, 19, 21–24]. Laparoscopic procedures have demonstrated decreased hospital length of stay, estimated blood loss, wound infection rates and time to adjuvant therapy with faster return of bowel function [19]. By extension, due to its similarity, robotic-assisted CRS/HIPEC is expected to have similar outcomes, however, there are few studies that have evaluated the role of robotics for PSM [18, 25–29]. We have demonstrated that at our center, patients who undergo robotic-assisted CRS/HIPEC have decreased hospital stay, estimated blood loss, transfusion requirement, and similar morbidity to their open counterparts. While the burden of disease was a main driver for selection of patients for robotic approach, the difference in PCI between the groups did not reach statistical significance. This may be due to the small number or due to differences in burden of disease that are not well reflected in the PCI score system. The underlying malignant type was similar between the groups. This shows that robotic-assisted surgery is applicable to many patients, when carefully selected. While long-term outcomes have not been considered in this analysis the important prognostic factor, completeness of cytoreduction, did not differ between open and robotic-assisted approach, suggesting that the oncologic integrity of the procedure remains intact. One concern that could be raised is the potential for lesions being missed during robotic operation which would result in false complete cytoreduction and possibly lead to recurrence.

Previous studies have shown that a decrease in perioperative blood transfusions is independently associated with improved perioperative and oncologic outcomes. Blood transfusions are associated with a dose-dependent effect on progression free survival and overall survival for patients with diffuse malignant peritoneal mesothelioma and pseudomyxoma peritonei that have undergone CRS/HIPEC [30]. This association remains true with gastrointestinal cancers as well. Canadian population data demonstrated that patients who underwent gastrointestinal cancer resection and received a red blood cell transfusion had an increased risk of all-cause and cancer-specific death relative to patients who did not [31]. Data from the U.S. HIPEC Collaborative Database have also shown an independent association between perioperative blood transfusion and postoperative risk of longer hospital stay, grade III or higher morbidity, and reoperation [32]. Selection of robotic-assisted CRS/HIPEC can improve perioperative and oncologic outcomes by limiting blood loss and the need for transfusions.

Robotic surgery can achieve a complete cytoreduction without a prohibitively long operative time, which can expand the minimally invasive approach to peritoneal surface malignancy. With improved visualization and dexterity, the platform has shown a decrease in conversion to open surgery than laparoscopic surgery for colectomies [33]. Theoretically this may translate to a higher likelihood of achieving a complete cytoreduction with the robotic platform. The largest retrospective series for laparoscopic CRS/HIPEC, of 215 patients, reported a median PCI of 3 [22]. Many of the laparoscopic CRS/HIPEC studies have suggested that a lower burden of disease, as defined by a PCI of less than 10, should be the target population for the minimally invasive approach [22, 24]. We have found that the robotic-assisted approach allows for a minimally invasive surgery for patients with a PCI score of greater than 10. Our data displayed no statistical difference in median PCI between the groups and our median robotic PCI was 10. This suggests that while high burden of disease may not be appropriate for minimally invasive approach, the PCI should not be a limitation to consider minimally invasive approaches. Further investigation of the capabilities to achieve a complete cytoreduction with a higher disease burden with the robotic approach is needed.

Like other minimally invasive studies, and in accordance with the Peritoneal Surface Oncology Group International recommendations, patient selection is critical for successful minimally invasive cytoreduction [15]. Patient preoperative evaluation includes the patient’s performance status, underlying tumor origin, and disease burden. Refinement of who is a candidate for CRS/HIPEC by evaluating the underlying disease and PCI has improved patient selection for those that benefit from cytoreduction [34]. Disease burden is evaluated with laparoscopic evaluation. Advances in diagnostic imaging may in the future allow for selection of patients for robotic approach. We have not defined an absolute PCI cutoff for selecting patients for robotic-assisted CRS/HIPEC, which is similar to other groups [19].

Surgeon comfort is another advantage to the platform which should be considered in this long, physically and technically challenging operation. Avoiding musculoskeletal injury and allowing surgeons to operate safely for more years is important for preservation of the workforce [35]. As more training programs are training surgeons in robotics, and as future platforms will come online, we expect surgical skills to increase for robotics allowing for expansion of minimally invasive approaches.

While not reviewed with this study, prior studies have displayed that equivalent oncologic outcomes can be achieved with careful patient selection, and laparoscopic approaches to CRS-HIPEC [15]. Further evaluation of our long-term data with consideration of performance status of the patient, disease-free survival, and overall survival is necessary with longer follow-up.

Our study has several limitations. Primarily, this is a single institution, single surgeon experience. Selection criteria for laparoscopic and robotic CRS/HIPEC have been proposed but have not been established. This leaves patient selection up to the surgeon and the best estimation for a complete cytoreduction for the patient, which may vary greatly between centers and surgeons. Due to the small sample size and single surgeon experience, extrapolation of our data to other centers needs further investigation.

We have demonstrated that robotic-assisted CRS/HIPEC offers select patients a shorter length of stay. Our study also highlights that important intraoperative and immediate perioperative outcomes are improved for these patients, such as a decrease in estimated blood loss and need for postoperative blood transfusions in the robotic group. PCI, completeness of cytoreduction scores, and rates of bowel anastomoses were not statistically different between the open and robotic groups. Further study is needed to better define patients who would be eligible to undergo robotic-assisted CRS/HIPEC and to determine if long-term oncologic outcomes, such as disease-free survival and overall survival, are equivalent between patients who undergo robotic versus open CRS/HIPEC.
